# Advances in surgical applications of growth factors for wound healing

**DOI:** 10.1186/s41038-019-0148-1

**Published:** 2019-04-05

**Authors:** Sho Yamakawa, Kenji Hayashida

**Affiliations:** Division of Plastic and Reconstructive Surgery, Shimane University Faculty of Medicine, 89-1 Enya-cho, Izumo, Shimane 693-8501 Japan

**Keywords:** Wound healing, Growth factor, Surgical application

## Abstract

Growth factors have recently gained clinical importance for wound management. Application of recombinant growth factors has been shown to mimic cell migration, proliferation, and differentiation *in vivo*, allowing for external modulation of the healing process. Perioperative drug delivery systems can enhance the biological activity of these growth factors, which have a very short *in vivo* half-life after topical administration. Although the basic mechanisms of these growth factors are well understood, most have yet to demonstrate a significant impact in animal studies or small-sized clinical trials. In this review, we emphasized currently approved growth factor therapies, including a sustained release system for growth factors, emerging therapies, and future research possibilities combined with surgical procedures. Approaches seeking to understand wound healing at a systemic level are currently ongoing. However, further research and consideration in surgery will be needed to provide definitive confirmation of the efficacy of growth factor therapies for intractable wounds.

## Background

Growth factors are endogenous signaling molecules that regulate cellular responses for wound healing process. These proteins are upregulated in response to tissue damage and are secreted by platelets, leukocytes, fibroblasts, and epithelial cells. Once growth factors are secreted, they act through autocrine, paracrine, or endocrine mechanisms by binding to membrane or cytoplasmic receptors. Binding to receptors results in a cascade of events that activate the cellular machinery to facilitate wound healing. Even at low concentrations, growth factors can have a marked impact on the wound microenvironment, leading to rapid increases in cell migration, proliferation, and differentiation [1]. *In vivo* and *in vitro* studies analyzing non-healing acute and chronic wounds have demonstrated de-regulation of several growth factors (e.g., platelet-derived growth factor (PDGF) [2], vascular endothelial growth factor (VEGF) [3], and fibroblast growth factor (FGF) [4]), suggesting a potential target for therapy, which has led to a robust interest in using exogenous growth factors and cytokines in the clinical setting to improve the outcomes of non-healing wounds. These evidences have led to a number of surgical applications where controlled drug delivery of human recombinant growth factors has great therapeutic potential [1]. Indeed, perioperative drug delivery of recombinant or exogenous growth factors is a routine adjunctive treatment in a lot of surgical fields, including burn surgery, oral surgery, orthopedic surgery, and plastic surgery [5–7]. However, recombinant or exogenous growth factors have limited clinical applications because they have a short *in vivo* half-life due to their low stability, restricted absorption rate through the skin around the wounds, and elimination by exudation before reaching the wounds after topical application [8].

Currently, with the advent of genetic engineering and advances in biological technology, there are many growth factors known to exert powerful effects for surgical use, including PDGF, VEGF, FGF, epidermal growth factor (EGF), keratinocyte growth factor (KGF), transforming growth factor beta (TGF-β), granulocyte-macrophage colony-stimulating factor (GM-CSF), and others [1]. Although the basic mechanisms of these growth factors are well understood, most have yet to demonstrate a significant impact in pre-clinical or small-sized trial. As there is a critical need for these new treatment options for the management of intractable wounds (e.g., pressure ulcers, venous leg ulcers, and diabetic foot ulcers), understanding how these growth factors may be utilized to optimize the wound microenvironment for healing is an exciting avenue of future research.

The purpose of this review is to outline the use of growth factors and release systems that prolong the bioactivity of growth factors as an alternative or adjunct to surgical treatment. In this review, we emphasized clinical outcome studies conducted on human subjects, with animal studies highlighted in the absence of clinical evidence for wound healing.

## Review

### Surgical debridement

Prior to the application of any growth factors, the contaminated wounds should be debrided meticulously and completely. Decreased angiogenesis, accumulation of devitalized tissue, increased proteases, hyperkeratotic tissue, and local infection around the wound are characteristics of chronic wounds, which prevent adequate cellular response to wound-healing stimuli [9]. It has been reported that wound bed preparation facilitates well-ordered restoration and regeneration of damaged tissue, and enhances the function of new therapies [9, 10].

**Fig. 1:**
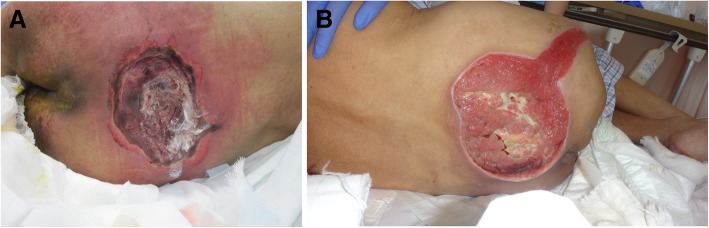
Pressure ulcer debridement. **a** This is a highly infected sacral pressure ulcer. Prior to the application of any growth factors, contaminated wounds should be meticulously and completely debrided. **b** This is the same pressure ulcer after debridement. Debridement of pressure ulcers is a safe and effective technique to make growth factor receptors respond to exogenous topical treatment

Surgical debridement is a promising approach of removing devitalized tissue from chronic wounds and a procedure to decrease bacterial contamination and infection while enabling the stimulation of wound contraction and epithelialization (Fig. 1a, b). Although the rationale for debridement seems logical, it is still unclear how to objectively determine the borders for surgical debridement. Currently, some molecular markers in patients with chronic wounds to guide surgical debridement have been reported, but the clinical evidence to support these hypotheses in enhancing wound healing is limited [11]. However, surgical debridement of chronic wounds is a safe and effective technique to make growth factor receptors respond to exogenous topical treatment. As the functions of growth factors are known to be dependent on their spatial distribution, controlling the delivery of growth factors temporally is important for their effective use as regenerative medicine in clinical settings [12]. The indications for surgical debridement include (1) removal of the source of sepsis, defined as systemic inflammatory response syndrome in the presence of infection; (2) decrease bacterial burden to reduce the probability of resistance to antibiotic treatment; (3) obtain accurate cultures taken after debridement from the tissue for systemic antibiotic treatment; and (4) stimulation of the wound bed to promote healing and prepare for flap surgery, skin grafting, or topical application of exogenous growth factors [13, 14].

### Growth factors: a promising approach for the treatment of intractable wounds

**Fig. 2:**
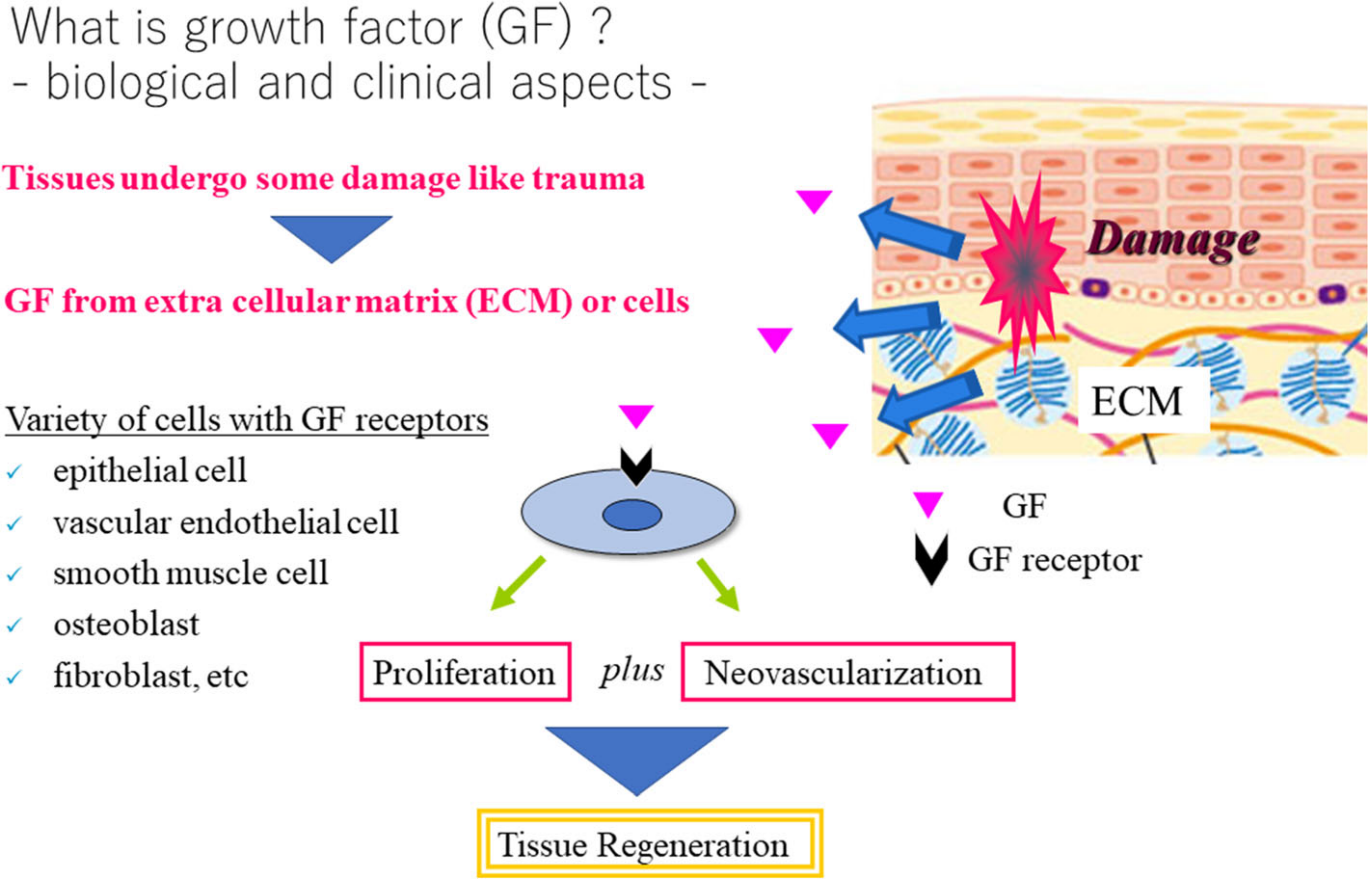
Biological and clinical aspect of growth factors

Topical administration of growth factors after debridement is a promising approach to enhance wound healing because of their deficiency or a noticeable deterioration of quality in chronic wounds (Fig. 2). Several approved medications including recombinant growth factors are available as preparations for external use in the form of solutions, ointments, creams, and gels.

Current medications containing growth factors require high doses and/or repeated administration over a long or short period of time, which could cause severe side effects including oncogenesis [15–17]. Such high-dose growth factors may increase the cost of therapy. Issues regarding safety and cost of the growth factor-loaded drug delivery systems (DDS) in clinical stages should be discussed to make growth factors widely accepted. However, some clinical studies of topically administrated growth factors have shown reliable evidence for therapeutic outcomes [18]. We review the recent clinical or animal studies using growth factors combined with surgical therapies for wound healing (Table 1).

**Table 1:** Representative growth factors and their applications for intractable wounds

Growth factors	Administration	Function	Effective wound type
PDGF	Topical	Regulate cell growth and division, chemoattractant for mesenchymal cells, angiogenesis	Diabetic foot ulcers
VEGF	Topical	Initiate angiogenesis; proliferation and migration of endothelial cells	Diabetic foot ulcers
EGF	Topical or intralesional injection	Stimulate proliferation and migration of keratinocytes; increase tensile strength of new skin	Burns, non-healing ulcers, and diabetic foot ulcers
bFGF	Topical	Stimulate proliferation, migration, and angiogenesis in injured skin	Pressure ulcers, venous ulcers, and burns
GM-CSF	Topical or subcutaneous injection	Recruit Langerhans cells, stimulate proliferation and differentiation	Non-healing wounds and venous ulcers

*PDGF* platelet-derived growth factor, *VEGF* vascular endothelial growth factor, *EGF* epidermal growth factor, *bFGF* basic fibroblast growth factor, *GM-CSF* granulocyte-macrophage colony-stimulating factor

#### PDGF family

##### PDGF

During the natural wound healing process, platelets are one of the first cell types to respond at or around the wound site, and pivotal to generating and initiating wound healing [1]. As mentioned above, no single exogenous agent can effectively facilitate all aspects of the wound-healing response [19]. Therefore, combination therapy with various treatments is required for successful cutaneous wound repair. Platelets have been used as a rich source of growth factors including PDGF. The PDGF are produced by platelets, macrophages, endothelial cells, fibroblasts, and keratinocytes [20]. PDGF has been found to regulate cell growth and division and play a role in angiogenesis [21, 22]. It is a potent mitogen and chemoattractant for mesenchymal cells [23].

PDGF is the first and only recombinant growth factor approved by the Food and Drug Administration (FDA) in the USA for topical administration and is used for the treatment of diabetic foot ulcers [20]. In a randomized controlled trial (RCT), a topical gel containing PDGF-BB (Regranex^®^) was compared with a placebo in 118 patients with non-healing diabetic ulcers enrolled from 10 different centers. Patients were treated for 20 weeks or until complete wound closure. Of the patients treated with PDGF, 48% healed compared with 25% of the patients treated with the placebo. A combined analysis of three additional clinical trials came to similar conclusions regarding the efficacy of PDGF-BB [24]. The results of these studies suggest that a daily dose of 100 μg/g PDGF-BB increases healing by as much as 39% compared with placebo. With an excellent safety profile and ease of administration, PDGF-BB should be considered for the treatment of diabetic foot ulcers, especially those unresponsive to standard care. However, of particular note, increased cancer risk has been reported in patients treated with more than three tubes of becaplermin (recombinant PDGF) [25]. So, we need for further research regarding the true correlation between cancer incidence rates and using becaplermin gel.

Topical applications of PDGF to pressure ulcers and venous ulcers have been attempted with minimal efficacy [26, 27]. The reasons for failed efficacy might be due to penetration of growth factors into the wound or age of the patients. Larger RCT are needed to test its efficacy in pressure ulcers and venous ulcers.

##### VEGF

The VEGF family is composed of VEGF-A, VEGF-B, VEGF-C, VEGF-D, VEGF-E, and placental growth factor [28]. Within this subset of proteins, VEGF-A is the best studied and has a notable role in initiating angiogenesis through the proliferation and migration of endothelial cells [29]. VEGF-A is secreted by platelets and macrophages in response to tissue injury in early wound healing [28]. In addition, hypoxia secondary to metabolic dysfunction is a major stimulus for the release of VEGF-A into the wound microenvironment [30]. Another clinical study shows that VEGF-A improves re-epithelialization of diabetic foot wounds associated with enhanced vessel formation [31].

Based on these improvements, VEGF165, a recombinant human-VEGF (rh-VEGF) gene carrying plasmid, has been just used in only patients with diabetic and ischemic wounds. Randomized controlled trials have been conducted on the efficacy of topical application of rh-VEGF in patients with neuropathic diabetic foot ulcers [32]. In the study, there were positive trends suggestive of potential signals of biological activity observed for incidence of complete ulcer healing (41.4% treatment vs 26.9% placebo), time to complete ulcer healing (32.5 days treatment vs 43.0 days placebo). Also, there is currently a phase II, double-blind randomized placebo-controlled study to assess the efficacy and/or safety of rh-VEGF treatment in patients with diabetic foot ulcer.

Compared with other growth factors, relatively few attempts have been made to use VEGF as an adjunctive treatment in wound healing. Early clinical studies on gene transfer had marginal success in delivering VEGF165 intramuscularly to treat non-healing, ischemic ulcers [33]. In animal models, the use of a protease-resistant VEGF-A has been proposed for use in the protease-rich microenvironment of chronic wounds [34]. Thus, despite promising studies in animal models, no topically based VEGF strategy has been reported. Instead, most therapies focusing on VEGF are anticancer treatments used to inhibit proliferation of tumor blood vessels [35].

#### EGF family

##### EGF

The EGF family of growth factors includes over a dozen proteins best characterized by EGF, heparin-binding EGF (HB-EGF), and transforming growth factor alpha (TGF-α). This subset of proteins has been extensively studied and is known to facilitate re-epithelialization by stimulating the proliferation and migration of keratinocytes [36]. Secondarily, the EGF family of proteins is responsible for increasing the tensile strength of new skin [37]. EGF proteins are secreted by fibroblasts, platelets, and macrophages and localize throughout the epidermis, particularly in the basal layer [38].

Within this family of growth factors, EGF has experienced the greatest use in human subjects. In an initial study conducted by Brown et al., EGF was used to supplement the healing of skin grafts following partial-thickness burns. Treatment with EGF reduced the time to complete wound re-epithelialization by 1.5 days compared with the control [39]. More recently, chronic wounds were found to exhibit decreased levels of EGF, providing rationale to deliver EGF to chronic, non-healing ulcers. Several studies evaluating the effects of EGF on diabetic foot ulcers concluded that treatment increases the incidence and rate of wound closure [40–42]. However, the challenge of using EGF or any other exogenous growth factor is that levels of matrix metalloproteinases are upregulated at sites of chronic inflammation. These proteases hinder wound healing by rapidly degrading growth factors or cytokines. Current treatment is thus limited by the lack of sophisticated delivery systems capable of providing sustained levels of EGF in addition to inhibiting its degradation.

Recently, to overcome the drawback, *in vivo *work using mice was reported [43]. The study was to utilize a novel payload comprising of Eudragit RL/RS 100 nanofibers carrying the bacterial inhibitor gentamicin sulfate (GS) in concert with human recombinant EGF. The Eudragit RL/RS 100 scaffolds with GS and EGF both showed more rapid wound closure rates as compared to the scaffolds with only GS and without EGF or to the treatment with pure GS ointment, preventing further bacterial infection challenges and promoting the wound healing process. This novel dual DDS allows for the synchronous release of GS and EGF and may serve as a faster and efficient therapy for the treatment of intractable ulcers.

Other members of the EGF family that have known roles in wound healing are HB-EGF and TGF-α. In animal studies, HB-EGF was transiently upregulated 2 to 4 days after wounding, indicating a role for this protein in early healing [44]. Moreover, application of HB-EGF to full-thickness wounds in mice increased proliferation and migration of keratinocytes at the wound bed [45].

Recombinant human EGF including Heberprot-P^®^, Regen-D™ 150, and Easyef^®^ is commercially available. Heberprot-P^®^ contains 75 μg of freeze-dried EGF and is administered intralesionally three times per week. A study of 20 diabetic patients who have foot ulcer showed full granulation response in all cases [46]. Intralesional injection into the deep wound layers has better availability, but pain at the injection site is a common complaint. Regen-D™ 150 is a gel containing 150 μg/g EGF that is applied topically twice a day. However, the effect of Regen-D™ 150 is still unknown. Easyef^®^ is a dermal solution spray indicated for diabetic foot ulcers. A prospective study reported that 21 of 89 patients showed improvement without EGF treatment, while complete healing of chronic diabetic foot ulcer was observed in 52 of 68 patients treated with EGF [47].

##### TGF-α

As a member of EGF family, TGF-α is a mitogenic polypeptide [48]. The function of TGF-α is similar to EGF. The main mechanism is inducing angiogenesis [1]. Similarly, studies conducted on mice have suggested a role for TGF-α in early re-epithelialization [49]. The data supports the concept that TGF-α plays a significant early role in wound epithelialization *in vivo*, but its deficit is compensated if accompanied by granulation tissue formation. However, other animal models about TGF-α in wound healing have not been tested yet. Additional basic or animal studies are needed to evaluate the function of TGF-α for wound healing.

#### FGF family

##### Basic FGF (bFGF)

The FGF family comprises over 20 isoforms known for their unique mechanism of action that involves binding to proteoglycans in the extracellular matrix (ECM) [50]. In general, the FGF proteins are potent mitogens that are instrumental in both normal growth and the wound healing process. Of these proteins, FGF-2, known as bFGF, is the best studied and has a confirmed role in the proliferation of both epithelial and mesenchymal cells as well as a possible role in angiogenesis [51].

Of the FGF family, bFGF has been the protein of choice for improving wound healing outcomes in humans. Robson et al. treated 61 pressure ulcers with bFGF, GM-CSF, or placebo. Ulcers treated with bFGF alone demonstrated the best healing with regard to wound closure and had elevated levels of bFGF, PDGF, and TGF-β1 in the wound fluid [52]. Similar findings were reported by Ohura et al., where treatment of pressure ulcers with exogenous bFGF resulted in accelerated healing [53]. Of note, administration of bFGF to diabetic foot ulcers provided no significant effects on healing [54]. FGF-10 has been successful in improving the healing rate of non-healing venous ulcers, albeit less extensively tested [55].

bFGF has also been used as an adjunctive treatment for burn wounds and fractures. Fu et al. did a prospective randomized double-blind multicenter trial to assess the effect of topical recombinant bFGF on burns [56]. They recruited 600 patients and described that the use of bFGF accelerated wound healing. Since burn wounds could be closed rapidly and the patient’s own skin soon became available for harvest and autografting, they concluded this growth factor had clinical benefits. Fiblast^®^ Spray is a commercially available recombinant human bFGF product indicated skin ulcers including leg ulcers and burn ulcers. Hayashida et al. reported that partial-thickness burn wounds in pediatric patients treated with bFGF exhibited accelerated healing, reduced scarring, and improved color matching with normal skin compared with controls up to half a year post-operatively [57, 58]. Akita et al. reported similar results in adult patients with burn wounds [59]. Although current results are promising, additional clinical trials are needed before FGF becomes widely accepted for the surgical use of cutaneous wounds.

##### Acidic FGF (aFGF)

Other FGF proteins intimately involved in wound healing are FGF-1, FGF-7, and FGF-10. FGF-1 is also known as aFGF. Acidic FGF is another classic and well-characterized member of the FGF family, and its structure, binding receptors, and biologic functions are similar to those of the bFGF. Ma et al. performed a randomized, multicenter, double-blind, and placebo-controlled clinical trial to assess the effect of topical aFGF on the healing of skin wounds [60]. In the study, 39 patients with deep-partial burns were included. The assessment results showed that the fully healed rate of the aFGF group was higher than that of the placebo group (53.85% vs 71.79%) in deep-partial burn wounds, and the mean healed time of the burn wounds treated by aFGF was significantly shorter than that of the placebo group (17.23 ± 0.53 vs 18.92 ± 0.49, *p* = 0.035). The results of their clinical trial showed that the wound healing process was faster and the healing time was also shortened in the aFGF-treated group. This suggests that aFGF has a potential therapeutic application for promoting healing of burn wounds. Although they obtained positive results of topical administration of aFGF for skin wound healing, long-term follow-up of clinical trial using aFGF is still expected before extensive clinical applications all over the world.

##### KGF

FGF-7, also known as KGF or palifermin, is an FGF protein. It preferentially affects epithelial cells and recruits fibroblasts in order to accelerate granulation tissue formation.

Staiano-Coico et al. and Danilenko et al. reported that KGF increased the rate of re-epithelialization and epidermal thickness in full- and partial-thickness wounds on porcine epidermis [61, 62].

FGF binding protein (FGF-BP), originally reported to bind and activate FGF-1 and FGF-2, also interacts with KGF and enhances the activity of low growth factor concentrations. Furthermore, expression of FGF-BP is increased following injury to murine skin, particularly in keratinocytes [63]. Thus, upregulation of FGF-BP following cutaneous injury may promote epithelial repair by stabilizing KGF and possibly providing protection from proteases in the wound environment. Use of this molecule, such as incorporating it into biomaterials, may augment the activity of KGF in wound healing applications [64]. So, these findings indicate that FGF-BP may be therapeutically explored for the enhancement of endogenous KGF activity at the wound site, and thus for the treatment of impaired wound healing.

Although current results are promising, additional clinical trials are needed before KGF becomes widely utilized for the surgical management of cutaneous wounds.

#### TGF-β

The TGF-β proteins are members of the TGF-β superfamily and exist as three functional isoforms: TGF-β1, TGF-β2, and TGF-β3. In the event of tissue injury, TGF-β is released into the wound microenvironment from storage sites in the ECM and secreted by macrophages, fibroblasts, and platelets [20]. In the early stages of wound healing, TGF-β has a reported role in modulating re-epithelialization, chemotaxis of leukocytes, and angiogenesis [65, 66]. However, the hallmark of TGF-β proteins is their ability to modulate wound contraction and scarring [65]. TGF-β1, 2, and 3 isoforms show a unique expression pattern spatially and temporally during cutaneous wound repair [67, 68]. Though TGF-β1 and TGF-β3 are largely homologous, they may exert opposing effects. In particular, one study suggested that, in contrast to TGF-β1, TGF-β3 may have an anti-fibrotic effect during wound healing and in different tissues: skin and mucosa [69].

Among the three isoforms, TGF-β1 is considered the most important in the process of wound healing [70]. TGF-β1-deficient mice develop massive inflammation, explaining why TGF-β1 has gained the attribute as an anti-inflammatory cytokine. Although this may be the case for adaptive immunity, for innate immunity, the influence of TGF-β may be dependent on the context [71]. However, contradictory results come from studies on TGF-β1 knockout mice or mice transgenically overexpressing TGF-β1. Depending on the system used and the age of the mice, TGF-β1 can both stimulate and protract wound re-epithelialization [72]. In fact, a recent animal study revealed that TGF-β1 gene transcription significantly correlates with the surgical vaginal and dermal wound closure rate [73]. Attempts to replicate this balance by antagonizing the effects of TGF-β1 *in vivo* have been successful in animals but not tested to a significant extent in humans [74, 75].

Conversely, delivery of TGF-β3 directly to the wound bed was an encouraging therapeutic option until 2011. Administration of TGF-β3 (Avotermin; Renovo, UK) significantly reduced scarring in a number of clinical trials before the drug failed to meet its endpoints in stage III clinical trials. While TGF-β1 promotes wound healing, it also may promote fibrosis when unchecked. In contrast, TGF-β3 may have an anti-fibrotic role in wound healing. Since both isoforms signal through the same receptors, it is still unknown how they have different biological behaviors toward the wound healing [68]. Although it is attractive in theory, TGF-β-based therapies have historically been disappointing [28]. An improved understanding of the TGF-β pathway coupled with novel approaches and delivery systems may be necessary before a TGF-β product secures FDA approval.

#### GM-CSF

GM-CSF is a cytokine found in the wound bed after acute injury that enables faster wound healing. Specifically, GM-CSF recruits Langerhans cells, stimulates local recruitment of inflammatory cells, advances myofibroblast differentiation to facilitate wound contraction, and mediates proliferation of the epidermis [76]. Recently, topical application of GM-CSF to refractory wounds was reported as effective in animal models, but systemic administration has no effects on wound healing [10].

Several studies about topical application of GM-CSF to chronic wounds were reviewed. Da Costa et al. [77] conducted a randomized controlled trial, with patients in the treatment arm receiving a perilesional injection of 200 μg or 400 μg of GM-CSF and the control arm receiving a placebo. After 13 weeks, complete healing was observed in 61% (11/18) receiving 400 μg and in 57% (12/21) receiving 200 μg. On the other hand, only 19% (4/21) healed completely in the control arm. In another trial with topical application of GM-CSF, 47 of 52 venous ulcers healed with an average healing period of 19 weeks (range 3–46) and a 90% overall healing rate. The re-ulceration rate over 1 year was 6% [76]. Khan et al. [78] compared these results with a study by Mayer et al. [79] who used standard compression therapy for venous ulcers and reported an all healing rate of 73% after 1 year. In Mayer’ study, their re-ulceration rate over 1 year was 30%. Therefore, topical application of GM-CSF to intractable wounds may be useful not only to speed up wound healing rate but also to prevent re-ulceration.

### Platelet-rich plasma (PRP) and platelet-rich fibrin (PRF) as scaffolds of growth factors

#### PRP

PRP was first described by Ferrari in 1987, where it was used to seal incisions made during open-heart surgery [80]. Since then, it has gained widespread use in a number of surgical fields for its ability to accelerate the healing of hard and soft tissues. By definition, PRP is a portion of the plasma fraction of autologous blood that contains an increased concentration of platelets [81]. The actual concentration of platelets varies based on the separation system but is generally in the range of 600,000 platelets/μL in a 5- to 7-mL volume [82].

PRP is produced by first withdrawing and centrifuging autologous blood in a buffered anticoagulant solution. This facilitates the separation of whole blood components by density and results in three layers: the erythrocyte layer, the platelet-rich buffy coat layer, and the plasma layer. The platelet-rich and plasma layers are aspirated and centrifuged a second time, resulting in a fraction of PRP that is applied to the surgical site in conjunction with a platelet activator such as calcium chloride or thrombin. Activated platelets immediately undergo degranulation, releasing alpha granules that contain an abundance of growth factors such as PDGF-αα, PDGF-αβ, PDGF-ββ, TGF-β, EGF, and VEGF [66, 83].

To date, PRP is the most frequently employed growth factor product during surgery [84, 85]. It has a particularly broad set of applications in dental surgery where it is used to improve wound healing of mucosal surfaces and bone. For example, PRP-treated bone grafts in sinus lift procedures exhibit increased osteogenesis and greater bone density 6 months post-operatively [86, 87], as well as accelerated healing of the overlying mucosa [88]. In tooth extraction, post-operative administration of PRP into the alveolar socket reduces pain and swelling 3 days post-operatively [89], improves the quality of hard and soft tissue healing [90, 91], and may decrease the incidence of hemorrhagic complications in patients taking anticoagulant medications [92]. Osseointegration of dental implants is also improved using PRP, suggesting potential therapeutic value in procedures designed around immediate loading [93, 94].

In contrast, there are conflicting results regarding the efficacy of PRP in periodontal surgery. A few studies have reported that adjunctive PRP may be beneficial for the treatment of intrabony or furcation defects [95, 96], but the majority found no effect or limited effects [97–99]. Similarly, application of PRP to a gingival graft did not improve periodontal outcomes after grafting [100].

Within the plastic surgery field, PRP has emerged as an effective treatment adjunct for cutaneous wounds and fat grafts. In cutaneous wounds, PRP appears to improve the rate of wound healing in healthy [101, 102] and diabetic patients [103–105]. In a study by Hom et al., full-thickness wounds made on volunteers were treated with or without PRP. Wounds treated with PRP exhibited accelerated wound closure and increased cellularity compared with controls, particularly in patients who achieved greater fold increases in platelet concentration relative to baseline levels [106]. Other observed benefits in PRP-treated wounds include decreased time to reconstructive surgery [101, 102], decreased length of hospital stay [102], and a decreased incidence of limb amputation regardless of underlying comorbidities [105, 107].

Although PRP has merited use in a number of surgical fields, much remains unknown regarding the optimal dose, platelet concentration, method of administration, and long-term outcomes in all fields of surgery [108]. Standardization of PRP to expand its clinical use also remains a problem as the varying concentrations of platelets, growth factors, and leukocytes are possibly responsible for conflicting study results. However, PRP is likely to gain popularity as an autologous, cost-effective preparation with minimal side effects.

#### PRF

Fibrin sealants have been used for several decades as hemostatic agents to achieve wound closure. Although initially successful, legal ramifications over concerns of viral transmission severely limited the distribution and use of these products [109]. PRF is a product derived from autologous blood with similar properties to the fibrin sealants [110]. Due to its autologous nature, PRF did not face the legal problems of its predecessors and has since been applied to a variety of surgical indications.

The methodology used to generate PRF is nearly identical to that of PRP. The main difference is that no anticoagulant or activator is used in the production of PRF, simplifying the process. In the absence of anticoagulants, the contact between the vial wall and the platelets during centrifugation stimulates the activation of the coagulation cascade. Fibrinogen present in the donor blood cross-links platelets and is converted to fibrin by endogenous thrombin. The final result is a platelet-charged fibrin clot that can be administered directly to the surgical site to stimulate wound healing and closure.

Although the fibrin clot plays a major role in hemostasis and secretion of growth factors, it does not achieve the same platelet concentration or levels of growth factors as PRP [111]. However, the fibrin clot can secondarily act as a three-dimensional scaffold to direct migration, proliferation, angiogenesis, and chemotaxis of inflammatory cells [112]. The size and shape of the formed clot can also be optimized for the surgical site, as recently demonstrated by Alio et al. in the use of an eye-shaped PRF clot for surgical repair of corneal perforation [113].

PRF has the greatest number of applications in dental surgery. For example, the use of adjunctive PRF in periodontal management of intrabony defects has resulted in improved osteogenesis, periodontal outcomes, and healing of mucosal surfaces compared with conventional treatment alone [114–116]. Of note, a study conducted by Pradeep et al. comparing adjunctive PRF and PRP in treating intrabony defects found that both therapies produced similar outcomes with regard to bone regeneration; however, the authors state their preference for PRF in clinical practice because of its simpler production protocol [117]. In another study, PRF in conjunction with surgical debridement was successful in facilitating bone regeneration in 15 of 15 patients presenting periapical lesions unresponsive to conventional endodontic treatment [118]. Within the oral and maxillofacial surgery fields, the use of adjunctive PRF may improve osteogenesis in sinus lift procedures, but available data is inconclusive [119, 120]. Like PRP, administration of PRF into the alveolar socket following tooth extraction may limit hemorrhage in patients on anticoagulant therapy [121].

The use of PRF has been limited in plastic surgery, with most studies using it as a therapeutic option to achieve wound closure [122, 123]. Patches or dressings incorporating patient-derived PRF have been described but have yet to be used on humans [124]. PRF has also been used in facial rejuvenation by Sclafani et al., where he observed new collagen deposition and angiogenesis 7 and 19 days post-treatment, respectively [125, 126]. Lastly, PRF has been applied in conjunction with fat grafts to improve survivability of the graft, with greater graft viability than that of PRP-treated grafts [127].

Although there are fewer surgical applications and less conclusive data for PRF compared with PRP, PRF is promising from a tissue engineering perspective for its properties as a scaffold with a complex three-dimensional architecture [128]. Coupled with its autologous nature, the versatility of PRF may lead to the discovery of novel therapeutics and delivery systems in the future.

### Current limitations of growth factors and future perspectives

Growth factors are well-established as critically important signaling molecules, but their use in surgery is currently limited. As of 2018, the greatest success has been autogenously derived growth factor preparations such as PRP and PRF. These formulations will likely continue to be successful and their role will expand within the surgical arena. Conversely, other growth factors with more limited roles, such as PDGF, TGF-β, or FGF, will flourish with advances in the fields of cell biology and immunology. The currently unremarkable results with the delivery of isolated growth factors indicate our incomplete understanding of how growth factors interact to guide wound healing.

Researchers of growth factor therapies focus on two key points. First, the effective use of growth factors is highly dependent on available delivery systems. Ongoing research has targeted this aspect of therapy with novel delivery platforms, such as polymer gels, coated dressings, chamber devices, and nanoparticles, described in recent reports [129–132]. Micro- and nanospheres are colloidal systems prepared using natural or synthetic materials, including poly lactic-co-glycolic acid (PLGA), alginate, gelatin, chitosan, and other polymer combinations [133–135]. Among them, PLGA is one of the most widely used polymers for GF entrapment in chronic wound therapy because it is biocompatible, biodegradable, less hydrophilic than other polymers, absorbs less water, and is thus slowly degraded, allowing for sustained drug release [136, 137]. Also, PLGA degradation produces lactate, which accelerates angiogenesis, activates pro-collagen factors, and recruits endothelial progenitor cells to the wound site. On this subject, Dong et al. [136] developed human recombinant EGF-loaded PLGA microspheres (MS) for chronic wound care that demonstrated an encapsulation efficiency of 85.6%. The *in vivo* studies showed that topical administration of human recombinant EGF-PLGA-MS to wounds enhanced the fibroblast proliferation rate and wound healing compared with free human recombinant EGF. In addition, the amount of proliferating cell nuclear antigen, which represents cell proliferation in the epidermis, was significantly greater in wounds treated with human recombinant EGF-PLGA-MS than in the control groups on days 7 and 14 after wound induction [136].

Using new bioinspired hydrogels with bFGF is also useful [138]. This study demonstrated that bioinspired hydrogels based on the chemical structure and nanomorphology of alga adhesive using gum arabic, pectin, and calcium combined with bFGF showed great promise for wound healing applications. The *in vivo* results showed that the bioinspired hydrogels with bFGF was able to significantly enhance cell proliferation, wound re-epithelialization, collagen deposition, and contraction without any toxicity and inflammation compared with the hydrogels without bFGF and commercially available wound healing products.

The second criticism of growth factor-based therapies is that sites of chronic inflammation generate complex microenvironments not amenable to treatment with a single growth factor [43]. The optimal therapeutic strategy is sustained delivery of growth factors that are able to withstand the abundance of proteases in the microenvironment. In addition, the proper growth factors must be secreted at the correct time and in precise concentrations to achieve favorable outcomes. Although growth factors mainly control interactions among cells or between cells and the ECM, wound care involving a single growth factor cannot completely manage the complex wound healing process, which is coordinated by the actions of multiple cell types including keratinocytes, fibroblasts, platelets, and other stromal cells [139]. This intricate interplay may be the reason for the great success of autologous products, such as PRP and PRF, where the identity and concentration of growth factor release are not under our control. Ito et al. reported that collagen/gelatin sponge impregnated with bFGF may be used as scaffolds with adipose tissue-derived stromal cells (ASCs) for adipogenesis [140]. The controlled release nature promotes bFGF-induced angiogenesis and ASCs proliferation. This modern technique is applicable for the reconstruction of volume contour deformities by surgical interventions of adipose tissues or trauma [140]. Wu et al. investigated the role of PDGF-AA in ASCs and endothelial progenitor cells enhancing wound healing [141]. In the study, they knocked down PDGF-AA expression in ASCs using the PDGF-AA short hairpin RNA technique and investigated the related molecular mechanism. The wound-healing assay of the study showed that transplantation of ASCs could enhance wound healing rate. The results showed that the PDGF-AA knockdown ASCs group had much less improvement of wound healing than other groups treated with wild-type ASCs in wound tissues. They concluded that PDGF-AA might play a vital role in ASCs enhancing wound healing, possibly by its effects on angiogenesis.

Effective translation of laboratory knowledge into clinical therapies will be necessary to better integrate growth factors into the framework of surgical management. One field of growing relevance is that of systems biology, which promises to aid us in understanding the dynamic network of signaling pathways. Approaches seeking to understand wound healing at a systems level are currently ongoing and may provide the paradigm shift needed to enhance our utilization of growth factors in surgery [142, 143]. For example, Garcia et al. conducted curative metatarsal bone surgery combined with intralesional administration of human recombinant EGF in neuropathic ulceration of the forefoot in patients with diabetes. There was a 2.1-fold shorter time for re-epithelization (healing), less recidivism, and a 2.3-fold decrease in lesions in the human recombinant EGF study group. The safety profile was appropriate based on the low frequency of complications and the light or moderate characteristics of the complications. Fever and shivering were more frequent in the human recombinant EGF-treated group [144].

To apply the growth factors, there are several procedures combined with surgical technique. Transplantation of skin fibroblasts into diabetic sheep with excisional wounds significantly increased the number of blood vessels and accelerated wound closure [145]. Cultured allogenic keratinocytes contributed for patients with venous ulcers or extensive burns with regard to clinical benefits [146, 147]. Keratinocytes in epidermal substitutes produce interleukin-1α and tumor necrosis factor-α, which synergistically mediate the secretion of wound-healing factors from fibroblasts in dermal substitutes [148]. As for epidermal or dermal substitutes, a bi-layered living cellular construct containing both keratinocytes and fibroblasts showed higher expression of cytokines and growth factors and greater endothelial network formation than did constructs containing only keratinocytes or fibroblasts [149].

Recently, one interesting research related to surgical sutures was reported [150]. The research introduced that standard surgical threads could be bio-activated with genetically modified microalgae to release both recombinant growth factors and oxygen directly into the wound site. They found this to be admissible as it makes the photosynthetic threads amenable to be stored as a “ready-to-use” or “off-the-shelf” biomaterial, thus facilitating its clinical translation. Although further researches are required to evaluate the efficacy and safety of this new technology *in vivo*, this represents the first step to create a new generation of surgical sutures with improved regenerative capabilities.

Gene therapy for the delivery of growth factors is also in an era of emerging treatment options for wound healing [151]. Shi et al. reported a combined gene transfer of VEGF-A and PDGF-B for diabetic foot ulcers in rats [152]. The aim of the study was to analyze whether the engineered growth factors based plasmid-loaded nanospheres could be upregulated in streptozotocin-induced diabetic rats and improve the wound healing. *In vivo*, the expression of VEGF-A and PDGF-B was significantly upregulated at full-thickness dorsal foot skin wounds, and the area of ulceration was significantly reduced following treatment with nanosphere/plasmid.

Growth factor has demonstrated potential in improving bone regeneration. In a study on foot and ankle surgery, Daniels et al. compared autogenous bone grafting with the use of an osteoconductive beta-tricalcium phosphate (β-TCP) scaffold enriched with PDGF-BB in patients undergoing hindfoot or ankle arthrodesis [153]. The prospective randomized controlled trial evaluated the efficacy and safety of PDGF-BB combined with an injectable β-TCP-collagen matrix. Seventy-five patients were randomized 5:1 for PDGF-BB/β-TCP-collagen (treatment, *n* = 63) or autograft (control, *n* = 12) and treated. They achieved clinical success in 57 of 63 (91%) PDGF-BB/β-TCP-collagen patients and in 120 of 154 (78%) autograft patients (*p* < 0.001) at 52 weeks. And, they concluded that the application of PDGF-BB/β-TCP-collagen was a safe and effective alternative for ankle and hindfoot fusions, eliminating the pain and morbidity associated with autograft bone harvesting. In the wound care arena, we also need to challenge prospective studies evaluating wound healing in combination with growth factors.

Although the clinical results of these growth factors are encouraging, most studies involved a small sample size and are disparate in measured endpoints. In addition, there are few references making a comparative study of the effects of each growth factor [154, 155]. A Cochrane systematic review inspected a heterogeneous group of trials that assessed 11 different growth factors for diabetic foot ulcers [156]. They found evidence suggesting that growth factors may increase the healing rate of diabetic foot ulcers. However, we are sure that the outcomes and conclusions are based on randomized clinical trials with high risk of systematic errors (bias). It is obvious that more clinical trials are required to assess the benefits and harms of growth factors in the management of diabetic foot ulcers.

## Conclusion

Proper surgical techniques and management remains paramount to achieving favorable outcomes. Further research which incorporates surgical procedures is needed to provide definitive confirmation of the efficacy of growth factor therapies for intractable wounds.

## Abbreviations

ASCs: Adipose tissue-derived stem cells
DDS: Drug delivery systems
EGF: Epidermal growth factor
FDA: Food and Drug Administration
FGF: Fibroblast growth factor
GM-CSF: Granulocyte-macrophage colony-stimulating factor
GS: Gentamicin sulfate
KGF: Keratinocyte growth factor
MS: Microspheres
PDGF: Platelet-derived growth factor
PLGA: Poly lactic-co-glycolic acid
PRF: Platelet-rich fibrin
PRP: Platelet-rich plasma
RCT: Randomized controlled trial
TGF-β: Transforming growth factor beta
VEGF: Vascular endothelial growth factor

## References

[1] Park JW, Hwang SR, Yoon IS. Advanced growth factor delivery systems in wound management and skin regeneration. Molecules. 2017;22:E1259. 10.3390/molecules22081259 PMC6152378 28749427

[2] Trengove NJ, Bielefeldt-Ohmann H, Stacey MC (2000). Mitogenic activity and cytokine levels in non-healing and healing chronic leg ulcers. Wound Repair Regen.

[3] Brown LF, Yeo KT, Berse B, Yeo TK, Senger DR, Dvorak HF (1992). Expression of vascular permeability factor (vascular endothelial growth factor) by epidermal keratinocytes during wound healing. J Exp Med.

[4] Powers CJ, McLeskey SW, Wellstein A (2000). Fibroblast growth factors, their receptors and signaling. Endocr Relat Cancer.

[5] Marck RE, Gardien KLM, Vlig M, Breederveld RS, Middelkoop E. Growth factor quantification of platelet-rich plasma in burn patients compared to matched healthy volunteers. Int J Mol Sci. 2019;20:E288. 10.3390/ijms20020288 PMC6358744 30642068

[6] Brooker JE, Camison LB, Bykowski MR, Hurley ET, Yerneni SS, Campbell PG (2019). Reconstruction of a calvarial wound complicated by infection: comparing the effects of biopatterned bone morphogenetic protein 2 and vascular endothelial growth factor. J Craniofac Surgery.

[7] Ho TC, Tsai SH, Yeh SI, Chen SL, Tung KY, Chien HY (2019). PEDF-derived peptide promotes tendon regeneration through its mitogenic effect on tendon stem/progenitor cells. Stem Cell Res Ther.

[8] Zhang S, Uludag H (2009). Nanoparticulate systems for growth factor delivery. Pharm Res.

[9] Brem H, Stojadinovic O, Diegelmann RF, Entero H, Lee B, Pastar I (2007). Molecular markers in patients with chronic wounds to guide surgical debridement. Mol Med.

[10] Barrientos S, Brem H, Stojadinovic O, Tomic-Canic M (2014). Clinical application of growth factors and cytokines in wound healing. Wound Repair Regen.

[11] Gordon KA, Lebrun EA, Tomic-Canic M, Kirsner RS (2012). The role of surgical debridement in healing of diabetic foot ulcers. Skinmed.

[12] Quatresooz P, Henry F, Paquet P, Pierard-Franchimont C, Harding K, Pierard GE (2003). Deciphering the impaired cytokine cascades in chronic leg ulcers (review). Int J Mol Med.

[13] Hess CT, Kirsner RS (2003). Orchestrating wound healing: assessing and preparing the wound bed. Adv Skin Wound Care.

[14] Hess CL, Howard MA, Attinger CE (2003). A review of mechanical adjuncts in wound healing: hydrotherapy, ultrasound, negative pressure therapy, hyperbaric oxygen, and electrostimulation. Ann Plast Surg.

[15] Price EW, Carnazza KE, Carlin SD, Cho A, Edwards KJ, Sevak KK (2017). ^89^Zr-DFO-AMG102 immuno-PET to determine local hepatocyte growth factor protein levels in tumors for enhanced patient selection. J Nucl Med.

[16] Yoshida K, Nakachi K, Imai K, Cologne JB, Niwa Y, Kusunoki Y (2009). Lung cancer susceptibility among atomic bomb survivors in relation to CA repeat number polymorphism of epidermal growth factor receptor gene and radiation dose. Carcinogenesis..

[17] Hayes CS, Defeo K, Dang H, Trempus CS, Morris RJ, Gilmour SK (2011). A prolonged and exaggerated wound response with elevated ODC activity mimics early tumor development. Carcinogenesis..

[18] Robson MC, Steed DL, Franz MG (2001). Wound healing: biologic features and approaches to maximize healing trajectories. Curr Probl Surg.

[19] Tabata Y (2005). Nanomaterials of drug delivery systems for tissue regeneration. Methods Mol Biol.

[20] Kiwanuka E, Junker J, Eriksson E (2012). Harnessing growth factors to influence wound healing. Clin Plast Surg.

[21] Salgado AJ, Coutinho OP, Reis RL (2004). Bone tissue engineering: state of the art and future trends. Macromol Biosci.

[22] Schilephake H (2002). Bone growth factors in maxillofacial skeletal reconstruction. Int J Oral Maxillofac Surg.

[23] Canalis E, McCarthy TL, Centrella M (1989). Effects of platelet-derived growth factor on bone formation in vitro. J Cell Physiol.

[24] Steed DL (2006). Clinical evaluation of recombinant human platelet-derived growth factor for the treatment of lower extremity ulcers. Plast Reconstr Surg.

[25] Papanas N, Maltezos E (2010). Benefit-risk assessment of becaplermin in the treatment of diabetic foot ulcers. Drug Saf.

[26] Rees RS, Robson MC, Smiell JM, Perry BH (1999). Becaplermin gel in the treatment of pressure ulcers: a phase II randomized, double-blind, placebo-controlled study. Wound Repair Regen.

[27] Margolis DJ, Morris LM, Papadopoulos M, Weinberg L, Filip JC, Lang SA (2009). Phase I study of H5.020CMV.PDGF-beta to treat venous leg ulcer disease. Mol Ther.

[28] Barrientos S, Stojadinovic O, Golinko MS, Brem H, Tomic-Canic M (2008). Growth factors and cytokines in wound healing. Wound Repair Regen.

[29] Senger DR, Ledbetter SR, Claffey KP, Papadopoulos-Sergiou A, Peruzzi CA, Detmar M (1996). Stimulation of endothelial cell migration by vascular permeability factor/vascular endothelial growth factor through cooperative mechanisms involving the alphavbeta3 integrin, osteopontin, and thrombin. Am J Pathol.

[30] Lokmic Z, Musyoka J, Hewitson TD, Darby IA (2012). Hypoxia and hypoxia signaling in tissue repair and fibrosis. Int Rev Cell Mol Biol.

[31] Galiano RD, Tepper OM, Pelo CR, Bhatt KA, Callaghan M, Bastidas N (2004). Topical vascular endothelial growth factor accelerates diabetic wound healing through increased angiogenesis and by mobilizing and recruiting bone marrow-derived cells. Am J Pathol.

[32] Hanft JR, Pollak RA, Barbul A, van Gils C, Kwon PS, Gray SM (2008). Phase I trial on the safety of topical rhVEGF on chronic neuropathic diabetic foot ulcers. J Wound Care.

[33] Baumgartner I, Pieczek A, Manor O, Blair R, Kearney M, Walsh K (1998). Constitutive expression of phVEGF165 after intramuscular gene transfer promotes collateral vessel development in patients with critical limb ischemia. Circulation.

[34] Mineur P, Colige AC, Deroanne CF, Dubail J, Kesteloot F, Habraken Y (2007). Newly identified biologically active and proteolysis-resistant VEGF-A isoform VEGF111 is induced by genotoxic agents. J Cell Biol.

[35] Chen HX, Cleck JN (2009). Adverse effects of anticancer agents that target the VEGF pathway. Nat Rev Clin Oncol.

[36] Nanney LB (1990). Epidermal and dermal effects of epidermal growth factor during wound repair. J Invest Dermatol.

[37] Brown GL, Curtsinger LJ, White M, Mitchell RO, Pietsch J, Nordquist R (1988). Acceleration of tensile strength of incisions treated with EGF and TGF-beta. Ann Surg.

[38] Nanney LB, Magid M, Stoscheck CM, King LE (1984). Comparison of epidermal growth factor binding and receptor distribution in normal human epidermis and epidermal appendages. J Invest Dermatol.

[39] Brown GL, Nanney LB, Griffen J, Cramer AB, Yancey JM, Curtsinger LJ (1989). Enhancement of wound healing by topical treatment with epidermal growth factor. N Engl J Med.

[40] Tsang MW, Wong WK, Hung CS, Lai KM, Tang W, Cheung EY (2003). Human epidermal growth factor enhances healing of diabetic foot ulcers. Diabetes Care.

[41] Viswanathan V (2006). A phase III study to evaluate the safety and efficacy of recombinant human epidermal growth factor (REGEN-D™ 150) in healing diabetic foot ulcers. Wounds.

[42] Fernandez-Montequin JI, Valenzuela-Silva CM, Diaz OG, Savigne W, Sancho-Soutelo N, Rivero-Fernandez F (2009). Intra-lesional injections of recombinant human epidermal growth factor promote granulation and healing in advanced diabetic foot ulcers: multicenter, randomised, placebo-controlled, double-blind study. Int Wound J.

[43] Dwivedi C, Pandey I, Pandey H, Patil S, Mishra SB, Pandey AC (2018). In vivo diabetic wound healing with nanofibrous scaffolds modified with gentamicin and recombinant human epidermal growth factor. J Biomed Mater Res A.

[44] Marikovsky M, Breuing K, Liu PY, Eriksson E, Higashiyama S, Farber P (1993). Appearance of heparin-binding EGF-like growth factor in wound fluid as a response to injury. Proc Natl Acad Sci U S A.

[45] Johnson NR, Wang Y (2013). Controlled delivery of heparin-binding EGF-like growth factor yields fast and comprehensive wound healing. J Control Release.

[46] Fernandez-Montequin JI, Betancourt BY, Leyva-Gonzalez G, Mola EL, Galan-Naranjo K, Ramirez-Navas M (2009). Intralesional administration of epidermal growth factor-based formulation (Heberprot-P) in chronic diabetic foot ulcer: treatment up to complete wound closure. Int Wound J.

[47] Hong JP, Jung HD, Kim YW (2006). Recombinant human epidermal growth factor (EGF) to enhance healing for diabetic foot ulcers. Ann Plast Surg.

[48] Ojeda SR, Ma YJ, Rage F (1997). The transforming growth factor alpha gene family is involved in the neuroendocrine control of mammalian puberty. Mol Psychiatry.

[49] Kim I, Mogford JE, Chao JD, Mustoe TA (2001). Wound epithelialization deficits in the transforming growth factor-alpha knockout mouse. Wound Repair Regen.

[50] Mohammadi M, Olsen SK, Ibrahimi OA (2005). Structural basis for fibroblast growth factor receptor activation. Cytokine Growth Factor Rev.

[51] Nakamizo S, Egawa G, Doi H, Natsuaki Y, Miyachi Y, Kabashima K (2013). Topical treatment with basic fibroblast growth factor promotes wound healing and barrier recovery induced by skin abrasion. Skin Pharmacol Physiol.

[52] Robson MC, Hill DP, Smith PD, Wang X, Meyer-Siegler K, Ko F (2000). Sequential cytokine therapy for pressure ulcers: clinical and mechanistic response. Ann Surg.

[53] Ohura T, Nakajo T, Moriguchi T, Oka H, Tachi M, Ohura N (2011). Clinical efficacy of basic fibroblast growth factor on pressure ulcers: case-control pairing study using a new evaluation method. Wound Repair Regen.

[54] Richard JL, Parer-Richard C, Daures JP, Clouet S, Vannereau D, Bringer J (1995). Effect of topical basic fibroblast growth factor on the healing of chronic diabetic neuropathic ulcer of the foot. A pilot, randomized, double-blind, placebo-controlled study. Diabetes Care.

[55] Robson MC, Phillips TJ, Falanga V, Odenheimer DJ, Parish LC, Jensen JL (2001). Randomized trial of topically applied repifermin (recombinant human keratinocyte growth factor-2) to accelerate wound healing in venous ulcers. Wound Repair Regen.

[56] Fu X, Shen Z, Chen Y, Xie J, Guo Z, Zhang M (1998). Randomised placebo-controlled trial of use of topical recombinant bovine basic fibroblast growth factor for second-degree burns. Lancet..

[57] Hayashida K, Akita S (2012). Quality of pediatric second-degree burn wound scars following the application of basic fibroblast growth factor: results of a randomized, controlled pilot study. Ostomy Wound Manage.

[58] Hayashida K, Akita S (2017). Surgical treatment algorithms for post-burn contractures. Burns & trauma.

[59] Akita S, Akino K, Yakabe A, Tanaka K, Anraku K, Yano H (2010). Basic fibroblast growth factor is beneficial for postoperative color uniformity in split-thickness skin grafting. Wound Repair Regen.

[60] Ma B, Cheng DS, Xia ZF, Ben DF, Lu W, Cao ZF (2007). Randomized, multicenter, double-blind, and placebo-controlled trial using topical recombinant human acidic fibroblast growth factor for deep partial-thickness burns and skin graft donor site. Wound Repair Regen.

[61] Staiano-Coico L, Krueger JG, Rubin JS, D'Limi S, Vallat VP, Valentino L (1993). Human keratinocyte growth factor effects in a porcine model of epidermal wound healing. J Exp Med.

[62] Danilenko DM, Ring BD, Tarpley JE, Morris B, Van GY, Morawiecki A (1995). Growth factors in porcine full and partial thickness burn repair. Differing targets and effects of keratinocyte growth factor, platelet-derived growth factor-BB, epidermal growth factor, and neu differentiation factor. Am J Pathol.

[63] Beer HD, Bittner M, Niklaus G, Munding C, Max N, Goppelt A (2005). The fibroblast growth factor binding protein is a novel interaction partner of FGF-7, FGF-10 and FGF-22 and regulates FGF activity: implications for epithelial repair. Oncogene..

[64] Finch PW, Mark Cross LJ, McAuley DF, Farrell CL (2013). Palifermin for the protection and regeneration of epithelial tissues following injury: new findings in basic research and pre-clinical models. J Cell Mol Med.

[65] Puolakkainen PA, Reed MJ, Gombotz WR, Twardzik DR, Abrass IB, Sage HE (1995). Acceleration of wound healing in aged rats by topical application of transforming growth factor-beta (1). Wound Repair Regen.

[66] Werner S, Grose R (2003). Regulation of wound healing by growth factors and cytokines. Physiol Rev.

[67] Le M, Naridze R, Morrison J, Biggs LC, Rhea L, Schutte BC, et al. Transforming growth factor beta 3 is required for excisional wound repair in vivo. PLoS One. 2012;7:e48040. 10.1371/journal.pone.0048040 PMC3482237 23110169

[68] Lichtman MK, Otero-Vinas M, Falanga V (2016). Transforming growth factor beta (TGF-beta) isoforms in wound healing and fibrosis. Wound Repair Regen.

[69] Chang Z, Kishimoto Y, Hasan A, Welham NV (2014). TGF-beta3 modulates the inflammatory environment and reduces scar formation following vocal fold mucosal injury in rats. Dis Model Mech.

[70] Kiritsi D, Nystrom A (2018). The role of TGFbeta in wound healing pathologies. Mech Ageing Dev.

[71] Li MO, Wan YY, Sanjabi S, Robertson AK, Flavell RA (2006). Transforming growth factor-beta regulation of immune responses. Annu Rev Immunol.

[72] Finnson KW, Arany PR, Philip A (2013). Transforming growth factor beta signaling in cutaneous wound healing: lessons learned from animal studies. Advances in wound care.

[73] Abramov Y, Hirsch E, Ilievski V, Goldberg RP, Botros SM, Sand PK (2013). Transforming growth factor beta 1 gene expression during vaginal vs cutaneous surgical woundexpression during vaginal vs cutaneous surgical wound healing in the rabbit. Int Urogynecol J.

[74] Reid RR, Roy N, Mogford JE, Zimmerman H, Lee C, Mustoe TA (2007). Reduction of hypertrophic scar via retroviral delivery of a dominant negative TGF-beta receptor II. J Plast Reconstr Aesthet Surg.

[75] Singer AJ, Huang SS, Huang JS, McClain SA, Romanov A, Rooney J (2009). A novel TGF-beta antagonist speeds reepithelialization and reduces scarring of partial thickness porcine burns. J Burn Care Res.

[76] Jaschke E, Zabernigg A, Gattringer C (1999). Recombinant human granulocyte-macrophage colony-stimulating factor applied locally in low doses enhances healing and prevents recurrence of chronic venous ulcers. Int J Dermatol.

[77] da Costa RM, Aniceto C, Jesus FM, Mendes M (1994). Quick healing of leg ulcers after molgramostim. Lancet.

[78] Khan MN, Davies CG (2006). Advances in the management of leg ulcers--the potential role of growth factors. Int Wound J.

[79] Mayer W, Jochmann W, Partsch H (1994). Varicose ulcer: healing in conservative therapy. A prospective study. Wiener medizinische Wochenschrift (1946).

[80] Ferrari M, Zia S, Valbonesi M, Henriquet F, Venere G, Spagnolo S (1987). A new technique for hemodilution, preparation of autologous platelet-rich plasma and intraoperative blood salvage in cardiac surgery. Int J Artif Organs.

[81] Marx RE (2004). Platelet-rich plasma: evidence to support its use. J Oral Maxillofac Surg.

[82] Castillo TN, Pouliot MA, Kim HJ, Dragoo JL (2011). Comparison of growth factor and platelet concentration from commercial platelet-rich plasma separation systems. Am J Sports Med.

[83] Lubkowska A, Dolegowska B, Banfi G (2012). Growth factor content in PRP and their applicability in medicine. J Biol Regul Homeost Agents.

[84] Tsai HC, Lehman CW, Chen CM (2019). Use of platelet-rich plasma and platelet-derived patches to treat chronic wounds. J Wound Care.

[85] Smith OJ, Jell G, Mosahebi A (2019). The use of fat grafting and platelet-rich plasma for wound healing: a review of the current evidence. Int Wound J.

[86] Poeschl PW, Ziya-Ghazvini F, Schicho K, Buchta C, Moser D, Seemann R (2012). Application of platelet-rich plasma for enhanced bone regeneration in grafted sinus. J Oral Maxillofac Surg.

[87] Khairy NM, Shendy EE, Askar NA, El-Rouby DH (2013). Effect of platelet rich plasma on bone regeneration in maxillary sinus augmentation (randomized clinical trial). Int J Oral Maxillofac Surg.

[88] Lindeboom JA, Mathura KR, Aartman IH, Kroon FH, Milstein DM, Ince C (2007). Influence of the application of platelet-enriched plasma in oral mucosal wound healing. Clin Oral Implants Res.

[89] Ogundipe OK, Ugboko VI, Owotade FJ (2011). Can autologous platelet-rich plasma gel enhance healing after surgical extraction of mandibular third molars?. J Oral Maxillofac Surg.

[90] Alissa R, Esposito M, Horner K, Oliver R (2010). The influence of platelet-rich plasma on the healing of extraction sockets: an explorative randomised clinical trial. Eur J Oral Implantol.

[91] Celio-Mariano R, de Melo WM, Carneiro-Avelino C (2012). Comparative radiographic evaluation of alveolar bone healing associated with autologous platelet-rich plasma after impacted mandibular third molar surgery. J Oral Maxillofac Surg.

[92] Della Valle A, Sammartino G, Marenzi G, Tia M, Espedito di Lauro A, Ferrari F (2003). Prevention of postoperative bleeding in anticoagulated patients undergoing oral surgery: use of platelet-rich plasma gel. J Oral Maxillofac Surg.

[93] Anitua EA (2006). Enhancement of osseointegration by generating a dynamic implant surface. J Oral Implantol.

[94] Anand U, Mehta DS (2012). Evaluation of immediately loaded dental implants bioactivated with platelet-rich plasma placed in the mandibular posterior region: a clinico-radiographic study. J Indian Soc Periodontol.

[95] Saini N, Sikri P, Gupta H (2011). Evaluation of the relative efficacy of autologous platelet-rich plasma in combination with beta-tricalcium phosphate alloplast versus an alloplast alone in the treatment of human periodontal infrabony defects: a clinical and radiological study. Indian J Dent Res.

[96] Kaushick BT, Jayakumar ND, Padmalatha O, Varghese S (2011). Treatment of human periodontal infrabony defects with hydroxyapatite + beta tricalcium phosphate bone graft alone and in combination with platelet rich plasma: a randomized clinical trial. Indian J Dent Res.

[97] Dori F, Nikolidakis D, Huszar T, Arweiler NB, Gera I, Sculean A (2008). Effect of platelet-rich plasma on the healing of intrabony defects treated with an enamel matrix protein derivative and a natural bone mineral. J Clin Periodontol.

[98] Dori F, Kovacs V, Arweiler NB, Huszar T, Gera I, Nikolidakis D (2009). Effect of platelet-rich plasma on the healing of intrabony defects treated with an anorganic bovine bone mineral: a pilot study. J Periodontol.

[99] Piemontese M, Aspriello SD, Rubini C, Ferrante L, Procaccini M (2008). Treatment of periodontal intrabony defects with demineralized freeze-dried bone allograft in combination with platelet-rich plasma: a comparative clinical trial. J Periodontol.

[100] Keceli HG, Sengun D, Berberoglu A, Karabulut E (2008). Use of platelet gel with connective tissue grafts for root coverage: a randomized-controlled trial. J Clin Periodontol.

[101] Kazakos K, Lyras DN, Verettas D, Tilkeridis K, Tryfonidis M (2009). The use of autologous PRP gel as an aid in the management of acute trauma wounds. Injury..

[102] Mazzucco L, Medici D, Serra M, Panizza R, Rivara G, Orecchia S (2004). The use of autologous platelet gel to treat difficult-to-heal wounds: a pilot study. Transfusion.

[103] Saad Setta H, Elshahat A, Elsherbiny K, Massoud K, Safe I (2011). Platelet-rich plasma versus platelet-poor plasma in the management of chronic diabetic foot ulcers: a comparative study. Int Wound J.

[104] Driver VR, Hanft J, Fylling CP, Beriou JM (2006). A prospective, randomized, controlled trial of autologous platelet-rich plasma gel for the treatment of diabetic foot ulcers. Ostomy Wound Manage.

[105] Sakata J, Sasaki S, Handa K, Uchino T, Sasaki T, Higashita R (2012). A retrospective, longitudinal study to evaluate healing lower extremity wounds in patients with diabetes mellitus and ischemia using standard protocols of care and platelet-rich plasma gel in a Japanese wound care program. Ostomy Wound Manage.

[106] Hom DB, Linzie BM, Huang TC (2007). The healing effects of autologous platelet gel on acute human skin wounds. Arch Facial Plast Surg.

[107] Glover JL, Weingarten MS, Buchbinder DS, Poucher RL, Deitrick GA, Fylling CP (1997). A 4-year outcome-based retrospective study of wound healing and limb salvage in patients with chronic wounds. Adv Wound Care.

[108] Middleton KK, Barro V, Muller B, Terada S, Fu FH (2012). Evaluation of the effects of platelet-rich plasma (PRP) therapy involved in the healing of sports-related soft tissue injuries. Iowa Orthop J.

[109] Jackson MR (2001). Fibrin sealants in surgical practice: an overview. Am J Surg.

[110] Shah R, MG T, Thomas R, Mehta DS.An update on the protocols and biologic actions of platelet rich fibrin in dentistry. Eur J Prosthodont Restor Dent. 2017;25:64-72. 10.1922/EJPRD_01690Shah09 28590091

[111] Kobayashi E, Fluckiger L, Fujioka-Kobayashi M, Sawada K, Sculean A, Schaller B (2016). Comparative release of growth factors from PRP, PRF, and advanced-PRF. Clin Oral Investig.

[112] Choukroun J, Diss A, Simonpieri A, Girard MO, Schoeffler C, Dohan SL (2006). Platelet-rich fibrin (PRF): a second-generation platelet concentrate. Part IV: clinical effects on tissue healing. Oral Surg Oral Med Oral Pathol Oral Radiol Endod.

[113] Alio JL, Rodriguez AE, Martinez LM, Rio AL (2013). Autologous fibrin membrane combined with solid platelet-rich plasma in the management of perforated corneal ulcers: a pilot study. JAMA Ophthalmol.

[114] Thorat M, Pradeep AR, Pallavi B (2011). Clinical effect of autologous platelet-rich fibrin in the treatment of intra-bony defects: a controlled clinical trial. J Clin Periodontol.

[115] Lekovic V, Milinkovic I, Aleksic Z, Jankovic S, Stankovic P, Kenney EB (2012). Platelet-rich fibrin and bovine porous bone mineral vs. platelet-rich fibrin in the treatment of intrabony periodontal defects. J Periodontal Res.

[116] Jankovic S, Aleksic Z, Klokkevold P, Lekovic V, Dimitrijevic B, Kenney EB (2012). Use of platelet-rich fibrin membrane following treatment of gingival recession: a randomized clinical trial. Int J Periodontics Restorative Dent.

[117] Pradeep AR, Rao NS, Agarwal E, Bajaj P, Kumari M, Naik SB (2012). Comparative evaluation of autologous platelet-rich fibrin and platelet-rich plasma in the treatment of 3-wall intrabony defects in chronic periodontitis: a randomized controlled clinical trial. J Periodontol.

[118] Singh S, Singh A, Singh R (2013). Application of PRF in surgical management of periapical lesions. Natl J Maxillofac Surg.

[119] Tatullo M, Marrelli M, Cassetta M, Pacifici A, Stefanelli LV, Scacco S (2012). Platelet rich fibrin (P.R.F.) in reconstructive surgery of atrophied maxillary bones: clinical and histological evaluations. Int J Med Sci.

[120] Gassling V, Purcz N, Braesen JH, Will M, Gierloff M, Behrens E (2013). Comparison of two different absorbable membranes for the coverage of lateral osteotomy sites in maxillary sinus augmentation: a preliminary study. J Craniomaxillofac Surg.

[121] Sammartino G, Dohan Ehrenfest DM, Carile F, Tia M, Bucci P (2011). Prevention of hemorrhagic complications after dental extractions into open heart surgery patients under anticoagulant therapy: the use of leukocyte- and platelet-rich fibrin. J Oral Implantol.

[122] Chignon-Sicard B, Georgiou CA, Fontas E, David S, Dumas P, Ihrai T (2012). Efficacy of leukocyte- and platelet-rich fibrin in wound healing: a randomized controlled clinical trial. Plast Reconstr Surg.

[123] O'Connell SM, Impeduglia T, Hessler K, Wang XJ, Carroll RJ, Dardik H (2008). Autologous platelet-rich fibrin matrix as cell therapy in the healing of chronic lower-extremity ulcers. Wound Repair Regen.

[124] Lundquist R, Holmstrom K, Clausen C, Jorgensen B, Karlsmark T (2013). Characteristics of an autologous leukocyte and platelet-rich fibrin patch intended for the treatment of recalcitrant wounds. Wound Repair Regen.

[125] Sclafani AP, McCormick SA (2012). Induction of dermal collagenesis, angiogenesis, and adipogenesis in human skin by injection of platelet-rich fibrin matrix. Arch Facial Plast Surg.

[126] Sclafani AP, Saman M (2012). Platelet-rich fibrin matrix for facial plastic surgery. Facial Plast Surg Clin North Am.

[127] Keyhan SO, Hemmat S, Badri AA, Abdeshahzadeh A, Khiabani K (2013). Use of platelet-rich fibrin and platelet-rich plasma in combination with fat graft: which is more effective during facial lipostructure?. J Oral Maxillofac Surg.

[128] Chien CS, Ho HO, Liang YC, Ko PH, Sheu MT, Chen CH (2012). Incorporation of exudates of human platelet-rich fibrin gel in biodegradable fibrin scaffolds for tissue engineering of cartilage. J Biomed Mater Res B Appl Biomater.

[129] Moura LI, Dias AM, Carvalho E, de Sousa HC (2013). Recent advances on the development of wound dressings for diabetic foot ulcer treatment--a review. Acta Biomater.

[130] Smith DM, Simon JK, Baker JR (2013). Applications of nanotechnology for immunology. Nat Rev Immunol.

[131] Demidova-Rice TN, Hamblin MR, Herman IM (2012). Acute and impaired wound healing: pathophysiology and current methods for drug delivery, part 2: role of growth factors in normal and pathological wound healing: therapeutic potential and methods of delivery. Adv Skin Wound Care.

[132] Vranckx JJ, Hoeller D, Velander PE, Theopold CF, Petrie N, Takedo A (2007). Cell suspension cultures of allogenic keratinocytes are efficient carriers for ex vivo gene transfer and accelerate the healing of full-thickness skin wounds by overexpression of human epidermal growth factor. Wound Repair Regen.

[133] Gainza G, Villullas S, Pedraz JL, Hernandez RM, Igartua M (2015). Advances in drug delivery systems (DDSs) to release growth factors for wound healing and skin regeneration. Nanomedicine.

[134] Degim Z (2008). Use of microparticulate systems to accelerate skin wound healing. J Drug Target.

[135] Ye M, Kim S, Park K (2010). Issues in long-term protein delivery using biodegradable microparticles. J Control Release.

[136] Dong X, Xu J, Wang W, Luo H, Liang X, Zhang L (2008). Repair effect of diabetic ulcers with recombinant human epidermal growth factor loaded by sustained-release microspheres. Sci China C Life Sci.

[137] Porporato PE, Payen VL, De Saedeleer CJ, Preat V, Thissen JP, Feron O (2012). Lactate stimulates angiogenesis and accelerates the healing of superficial and ischemic wounds in mice. Angiogenesis.

[138] Zhang X, Kang X, Jin L, Bai J, Liu W, Wang Z (2018). Stimulation of wound healing using bioinspired hydrogels with basic fibroblast growth factor (bFGF). Int J Nanomedicine.

[139] Akita S, Hayashida K, Takaki S, Kawakami Y, Oyama T, Ohjimi H (2017). The neck burn scar contracture: a concept of effective treatment. Burns Trauma.

[140] Ito R, Morimoto N, Liem PH, Nakamura Y, Kawai K, Taira T (2014). Adipogenesis using human adipose tissue-derived stromal cells combined with a collagen/gelatin sponge sustaining release of basic fibroblast growth factor. J Tissue Eng Regen Med.

[141] Wu LW, Chen WL, Huang SM, Chan JY. Platelet-derived growth factor-AA is a substantial factor in the ability of adipose-derived stem cells and endothelial progenitor cells to enhance wound healing. FASEB J. 2019;33:2388-95. 10.1096/fj.201800658R 30265575

[142] Menke NB, Cain JW, Reynolds A, Chan DM, Segal RA, Witten TM (2010). An in silico approach to the analysis of acute wound healing. Wound Repair Regen.

[143] An G, Faeder J, Vodovotz Y (2008). Translational systems biology: introduction of an engineering approach to the pathophysiology of the burn patient. J Burn Care Res.

[144] Garcia Herrera AL, Febles Sanabria RJ, Acosta Cabadilla LLA, Moliner CM (2017). Curative metatarsal bone surgery combined with intralesional administration of recombinant human epidermal growth factor in diabetic neuropathic ulceration of the forefoot: a prospective, open, uncontrolled, nonrandomized, observational study. Current therapeutic research, clinical and experimental.

[145] Kazemi-Darabadi S, Sarrafzadeh-Rezaei F, Farshid AA, Dalir-Naghadeh B (2014). Allogenous skin fibroblast transplantation enhances excisional wound healing following alloxan diabetes in sheep, a randomized controlled trial. Int J Surg.

[146] Leigh IM, Purkis PE, Navsaria HA, Phillips TJ (1987). Treatment of chronic venous ulcers with sheets of cultured allogenic keratinocytes. Br J Dermatol.

[147] Auxenfans C, Shipkov H, Bach C, Catherine Z, Lacroix P, Bertin-Maghit M (2014). Cultured allogenic keratinocytes for extensive burns: a retrospective study over 15 years. Burns..

[148] Spiekstra SW, Breetveld M, Rustemeyer T, Scheper RJ, Gibbs S (2007). Wound-healing factors secreted by epidermal keratinocytes and dermal fibroblasts in skin substitutes. Wound Repair Regen.

[149] Wojtowicz AM, Oliveira S, Carlson MW, Zawadzka A, Rousseau CF, Baksh D (2014). The importance of both fibroblasts and keratinocytes in a bilayered living cellular construct used in wound healing. Wound Repair Regen.

[150] Centeno-Cerdas C, Jarquin-Cordero M, Chavez MN, Hopfner U, Holmes C, Schmauss D (2018). Development of photosynthetic sutures for the local delivery of oxygen and recombinant growth factors in wounds. Acta Biomater.

[151] Desmet CM, Preat V, Gallez B (2018). Nanomedicines and gene therapy for the delivery of growth factors to improve perfusion and oxygenation in wound healing. Adv Drug Deliv Rev.

[152] Shi R, Lian W, Han S, Cao C, Jin Y, Yuan Y (2018). Nanosphere-mediated co-delivery of VEGF-A and PDGF-B genes for accelerating diabetic foot ulcers healing in rats. Gene Ther.

[153] Daniels TR, Younger AS, Penner MJ, Wing KJ, Le IL, Russell IS (2015). Prospective randomized controlled trial of hindfoot and ankle fusions treated with rhPDGF-BB in combination with a beta-TCP-collagen matrix. Foot Ankle Int.

[154] Tan Y, Xiao J, Huang Z, Xiao Y, Lin S, Jin L (2008). Comparison of the therapeutic effects recombinant human acidic and basic fibroblast growth factors in wound healing in diabetic patients. J Health Sci.

[155] Fu XB, Sun TZ, Wang YP (1999). Comparative study of epidermal growth factor and basic fibroblast growth factor on wound healing. Zhongguo Xiu Fu Chong Jian Wai Ke Za Zhi.

[156] Marti-Carvajal AJ, Gluud C, Nicola S, Simancas-Racines D, Reveiz L, Oliva P, et al. Growth factors for treating diabetic foot ulcers. Cochrane Database Syst Rev. 2015;28:CD008548. 10.1002/14651858.CD008548.pub2 PMC8665376 26509249

